# Age differences in what-if thinking from midlife onwards: Prefrontal contribution and implications for emotional health in late life

**DOI:** 10.1007/s11357-025-01928-8

**Published:** 2025-10-07

**Authors:** Riccardo M. Galli, Friederike Thams, Bernd Löwe, Bastian Cheng, Götz Thomalla, Max Bieder, Elina L. Petersen, Christian Büchel, Stefanie Brassen

**Affiliations:** 1 Department of Systems Neuroscience, University Medical Centre Hamburg-Eppendorf, Hamburg, Germany; 2 Department of Psychosomatic Medicine and Psychotherapy, University Medical Centre Hamburg-Eppendorf, Hamburg, Germany; 3 Department of Neurology, University Medical Centre Hamburg-Eppendorf, Hamburg, Germany; 4 Department of Neuroscience, University of Geneva, Geneva, Switzerland; 5 Hamburg City Health Study, University Medical Center Hamburg-Eppendorf, Hamburg, Germany

**Keywords:** Regret, Emotional resilience, Successful aging, Depression, Counterfactual thinking

## Abstract

Reduced counterfactual (“What-If”) thinking (CFT) in late life is believed to reflect successful adaptation to limited resources for overcoming missed opportunities, thereby preventing feelings of regret. We propose that this adaptation becomes critical from midlife onwards, affects emotional regulation, and is mediated by the preserved functionality of the ventromedial prefrontal cortex (vmPFC). To test this, an epidemiological sample from the Hamburg City Health Study (N = 494), aged 48 to 75, completed a sequential risk-taking task presenting missed opportunities to induce CFT, and was assessed for life satisfaction, depressive symptoms, emotion regulation, and resting-state functional connectivity of the vmPFC. In line with the ‘paradox of aging,’ our cross-sectional data indicate a positive linear relationship between age and life satisfaction from midlife onward, alongside a negative association between age and depressive symptoms. Crucially, counterfactual thinking (CFT) was also lower in older compared to younger adults; however, reduced CFT in later life was restricted to individuals free of significant depressive symptoms, suggesting that a lack of disengagement from regrets specifically in older age enhances the risk for depression. Mediation models suggest that reduced what-if-thinking improves emotional well-being in late life by facilitating positive reappraisal and depends on preserved vmPFC connectivity with the dorsolateral PFC. Our findings underscore the increasing role of CFT and intra-prefrontal connectivity in emotional adaptation in the second half of life. Equipping middle-aged adults with strategies to address missed opportunities may significantly enhance resilience in aging populations.

**Graphical Abstract:**

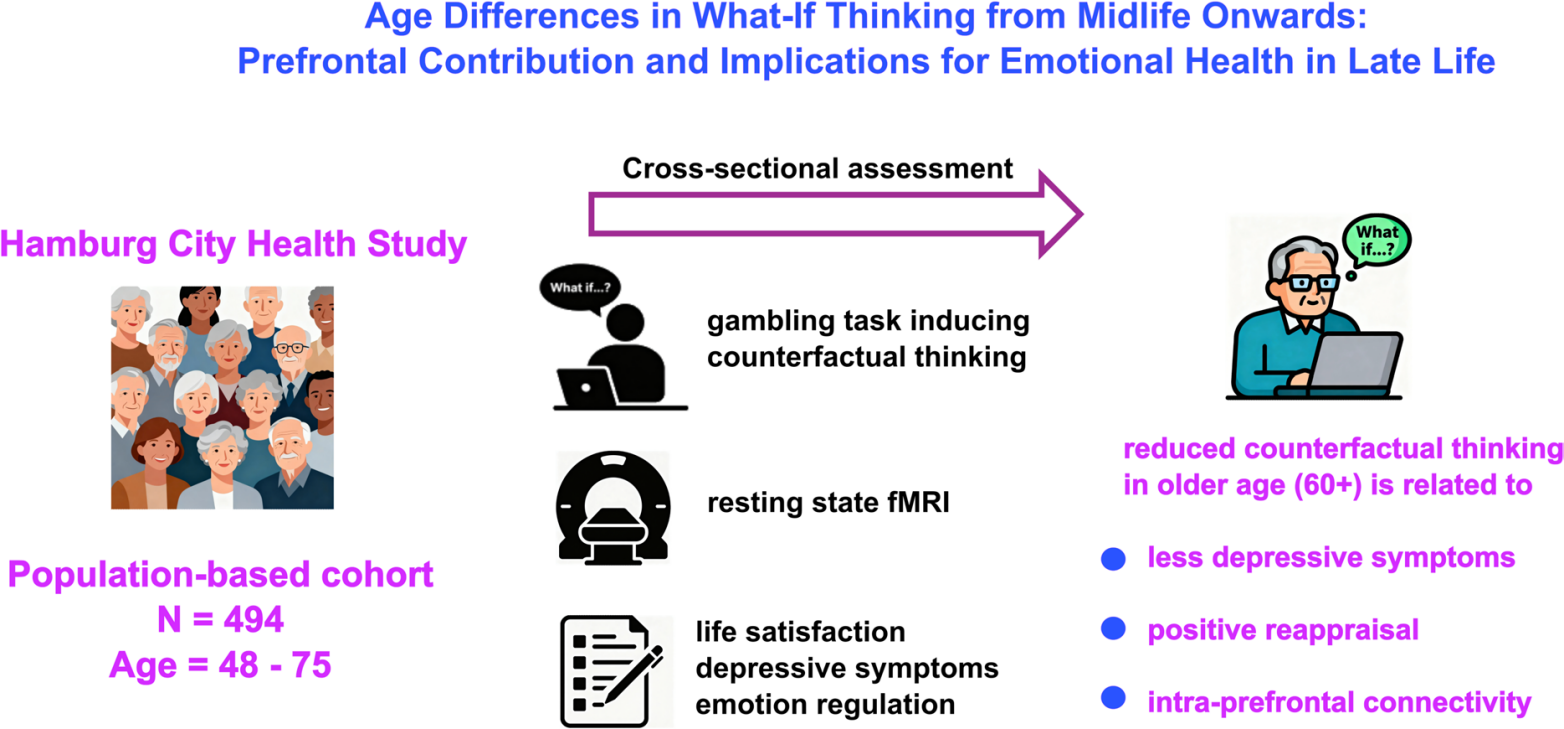

**Supplementary Information:**

The online version contains supplementary material available at 10.1007/s11357-025-01928-8.

## Introduction

Counterfactual thinking (CFT) describes the creation of alternatives to reality when we think “if only…” or “what if…” and thus imagine how things could have turned out differently. Upward counterfactuals, i.e., when people imagine better alternative states, such as having made the right choices, can evoke feelings of regret and predict depression, especially when self-blaming for these decisions [1–3]. This risk may become particularly relevant in older age [4], where people often review their past [5], but less opportunities to undo consequences of regrettable behavior remain [6, 7]. Indeed, a youth-like level of counterfactual thinking (CFT) in older age is related to depression [8]. In contrast, non-depressed older adults exhibit fewer responses to counterfactuals and lower ruminative thinking compared to their depressed peers and younger individuals [8, 9]. This suggests that the ability to actively reduce CFT is an important resilience factor for emotional well-being in aging.

The downregulation of counterfactual thoughts may have a significant impact on how individuals manage everyday challenges [2, 10]. Healthy older adults tend to engage in positive reappraisal when faced with negative situations in their daily lives [11]. Reduced counterfactual thinking may promote these adaptive coping strategies by aiding older adults to discover positive meanings in their negative experiences, rather than ruminating on missed opportunities and, consequently, avoiding feelings of regret. Understanding this specific mechanism of age-adapted regulatory styles can help identify malleable targets and critical time-windows for interventions aimed at preserving emotional well-being in later life.

While most studies on resilience and healthy aging have utilized extreme age-group designs, effective adaptation likely begins in midlife, when psychological and socioeconomic challenges intersect with physical, cognitive, and neural changes [12–16]. Emotional well-being often improves after age 50 [17, 18] but it is unclear whether changes in CFT follow similar trajectories.

Imaging findings indicate that reduced CFT is mediated by medial prefrontal regulatory processes, enabling older adults to focus on positive outcomes rather than ruminating on missed opportunities [8]. The ventromedial prefrontal cortex (vmPFC) plays a crucial role in emotional aging [19, 20] and facilitates a shift toward positive processing [21–23]. It is suggested that relatively preserved vmPFC functioning in aging allows for less resource-intensive implicit and habitual regulation [16, 24, 25], compensating for declines in cognitive and lateral prefrontal functioning [20]. Alternatively, some argue that increased vmPFC activity and decreased amygdala engagement are merely epiphenomena of the aging process [26, 27]. To ultimately investigate this question, neural aging and adaptation processes must be disentangled.

In this study, we aimed to enhance our understanding of CFT in the second half of life, with the overarching goal of identifying early targets for preventing mood disorders in older adults. To this end, we analyzed a large epidemiological dataset comprising 494 participants aged 48 to 75 years, using an established paradigm sensitive to CFT (the “devil task,” [8, 28]). Furthermore, we assessed resting-state vmPFC connectivity, emotional status, and preferences for positive reappraisal versus rumination among all participants. This approach enabled us to, first, evaluate the replicability of an enhanced well-being and a positivity focus from midlife onward (“paradox of aging”, [29]). Secondly, by leveraging the continuous age range in our sample, we could test whether previous findings from young-old comparisons indeed result from gradual adaptation processes across the lifespan. Thirdly, and most importantly, our design enabled the integration of age differences in experimental CFT data with emotional and neural measures, allowing us to test mechanistic assumptions about the conditions and consequences of adapted CFT.

## Results

### Sample characteristics

Data were collected from the Hamburg City Health Study (HCHS; www.hchs.hamburg), a single-center prospective observational cohort study aimed at gaining insights into disease development and survivorship among adults aged 48 to 75. The final sample comprised 494 middle- to old-aged adults in whom valid experimental data from the devil task, measures of positive and negative aspects of emotional well-being (i.e., life satisfaction and depressive symptoms) as well as rsfMRI data were available. Data exclusions are summarized in the supplementary study flow chart (Supplementary Fig. 1). For characterization purposes the sample was divided into 5 age subgroups. The groups did not significantly differ in size or gender distribution (all *P* > 0.56; see Table 1 for details). The local ethics committee approved the study (Ethikkommission der Ärztekammer Hamburg, PV5131). All participants gave written informed consent. The study has been pre-registered (https://osf.io/gzbqv).

**Table 1:** Sample characteristics

			Age Group				
Sample Characteristics		Full sample	48–53	54–59	60–64	65–68	69–75
Sample size	*n*	494	95	100	101	93	105
Female	*%*	45.7	45.3	53.0	41.6	44.1	44.8
Male	*%*	54.3	54.7	47.0	58.4	55.9	55.2
Age (in years)	*M* *(SD)*	61.7 (7.6)	50.9 (1.5)	56.4 (1.8)	62.2 (1.5)	66.6 (1.1)	71.8 (1.9)
MMSE	*M* *(SD)*	28.1 (1.4)	28.2 (1.4)	28.4 (1.3)	28.2 (1.4)	28.1 (1.4)	27.6 (1.5)
In Partnership	*% yes*	73.7	71.6	70.0	69.3	86.0	72.4

MMSE = Mini-Mental State Examination.

### Age and emotional well-being

Regression models showed a significant higher life satisfaction, as assessed via a 0- to 10-steps ladder [18], with age (β = 0.11, *P* = 0.014). This effect persisted when adjusting for relevant covariates (gender, partnership, global cognition in MMSE; β = 0.12, *P* = 0.007). Similarly, age was negatively associated with depressive symptoms as assessed with the PHQ-9 without (β = −0.14*, P* < 0.002) and with adjusting for covariates (β = −0.15, *P* < 0.001).

### Emotion regulation styles and well-being

**Fig. 1:**
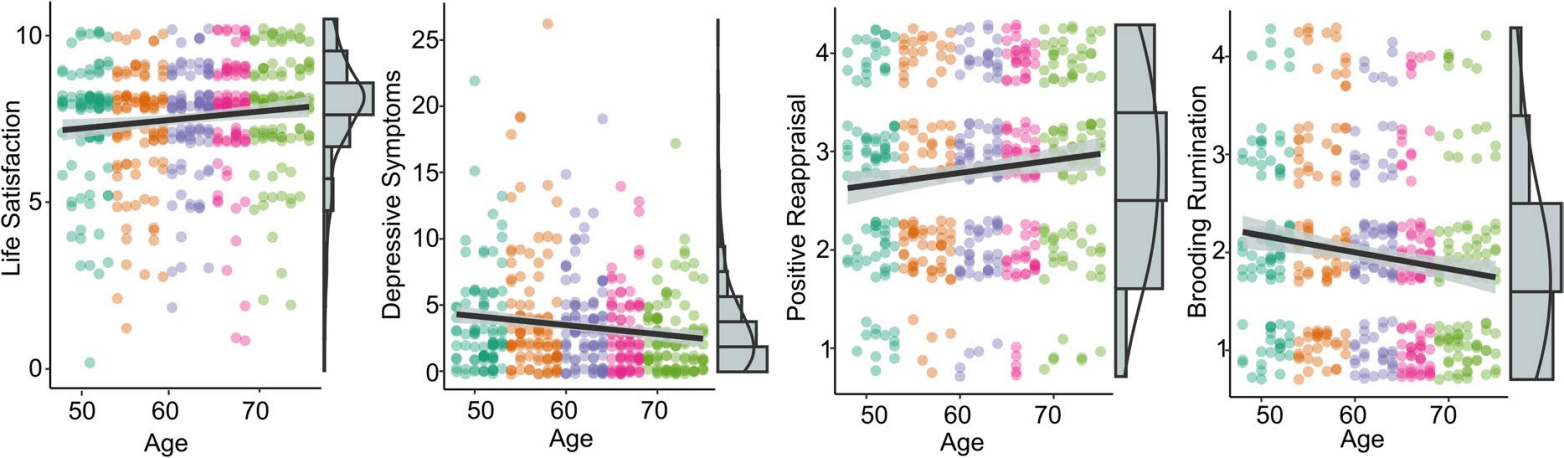
Age profiles of emotional measures. There was a positive relation of age with life satisfaction and positive reappraisal, while depressive symptoms (PHQ-9 score) and ruminative thinking were negatively associated with age. Jittering was used for visualization purposes

Participants answered how often they use a ruminative or positive reappraisal coping style in daily life on a 4-point scale (1 = never, 4 = always). Due to missing data, the sample size was slightly reduced (N = 483). Regression models demonstrate that brooding rumination as general regulation style was less often used in older age (β = −0.14, *P* = 0.002), while positive reappraisal was more frequently used (β = 0.10, *P* = 0.04, Fig. 1).

**Fig. 2:**
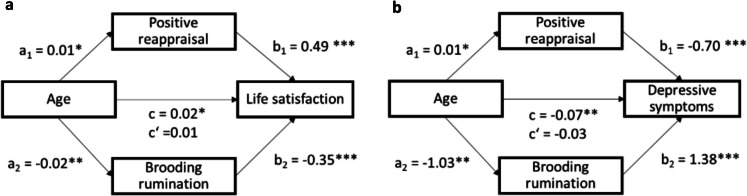
Mediation of age-effects on emotional well-being by preferred coping strategies. Age-related higher usage of positive reappraisal and lower usage of brooding rumination independently mediate positive age-effects on life satisfaction (a, model 1) and depressive symptoms (b, model 2). *P < 0.05, ** *P* < 0.005, *** *P* < 0.001

We then tested the mediating effect of regulation styles on age-effects in measures of emotional well-being. Parallel mediation analyses demonstrated a full mediation of positive age-effects on both life satisfaction (Fig. 2a; model 1) and depressive symptoms (Fig. 2b, model 2) by an independent impact of increased positive reappraisal (model 1: standardized *a*_*1*_*b*_*1*_ = 0.02, 95%-CI[0.002 0.050], model 2: *a*_*1*_*b*_*1*_ = −0.02, 95%-CI[−0.036 −0.0003]) and decreased brooding rumination (model 1: *a*_*2*_*b*_*2*_ = 0.02, 95%-CI[0.007 0.050]; model 2: *a*_*2*_*b*_*2*_ = −0.05, 95%-CI[−0.082 −0.018]).

### Counterfactual thinking and emotional well-being

**Fig. 3:**
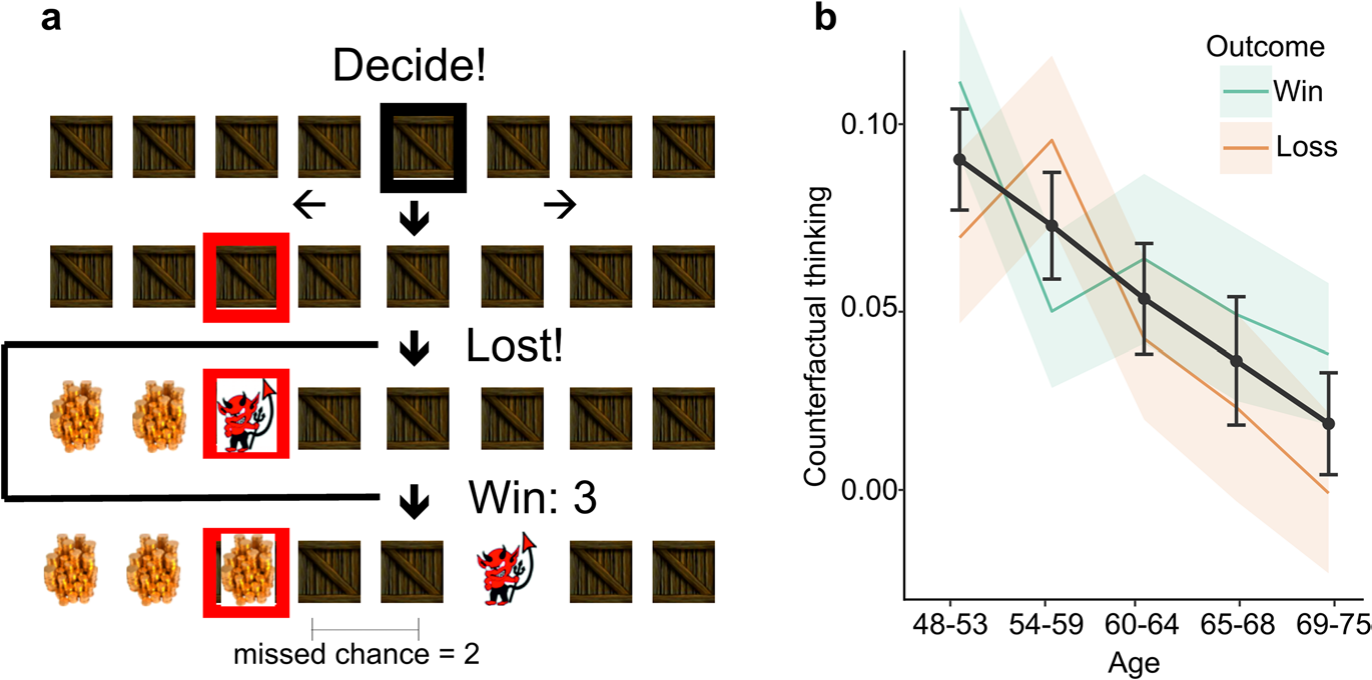
Outline of the paradigm (**a**) and age-effects on CFT for win and loss trials separately and across all trials (**b**)

All participants performed a modified sequential risk-taking task ('devil task', Fig. 3a) on a computer. Each trial began with eight closed boxes, with seven containing gold and one containing a randomly placed devil. Participants chose how many boxes to open (1 gold = 1 win point). All selected boxes were then opened, including the devil box. In loss trials, the devil was in a box preceding or equal to the stop box, whereas in win trials, the devil was in a later box. The difference between the stop box and the devil box is considered the 'missed chance'.

As in our previous study [8], general risk-taking did not differ between age groups (F(4,489) = 1.17, *P* = 0.32; mean choice [SD]: 48–53 = 4.01 [0.83]; 54–59 = 3.87 [0.77]; 60–64 = 3.86 [0.77]; 65–68 = 3.81 [0.87]; 69–75 = 3.80 [0.82]). Likewise, replicating previous findings [8, 28], our paradigm induced CFT across participants, i.e. missed opportunities in the current trial predicted risk-taking in the subsequent trial (*t*(494) = 6.04, *P* < 0.001), even though the “devil” was randomly distributed and the consideration of missed opportunities was irrelevant for outcomes in the independent trials. In this modified version of the task, we were now also able to observe a relationship between missed opportunities in loss trials and subsequent risk taking (*t*(494) = 4.04, *P* < 0.001), due to lower risk-taking after trials with near misses to win (near-wins). Both indicators of (upward) counterfactual thinking (“I could have won [more]”) were significantly reduced with increasing age (β_ct_win_ = −0.09, p = 0.042, see Eq. 1; β_ct_lost_ = −0.14, *P* = 0.003, see Eq. 2), i.e., CFT gradually approached zero. Since both parameters showed similar age-effects with no specific interactions (rmANCOVA with the two CFT levels and the covariate age: age: F(1,492) = 14.07, *P* < 0.001; CFT_dimension x age: F(1,492) = 0.54, *P* = 0.46), we aggregated both dimensions into a single CFT indicator, which further increased the effect size of age-effects (β = −0.17, *P* < 0.001; Fig. 3b).

**Fig. 4:**
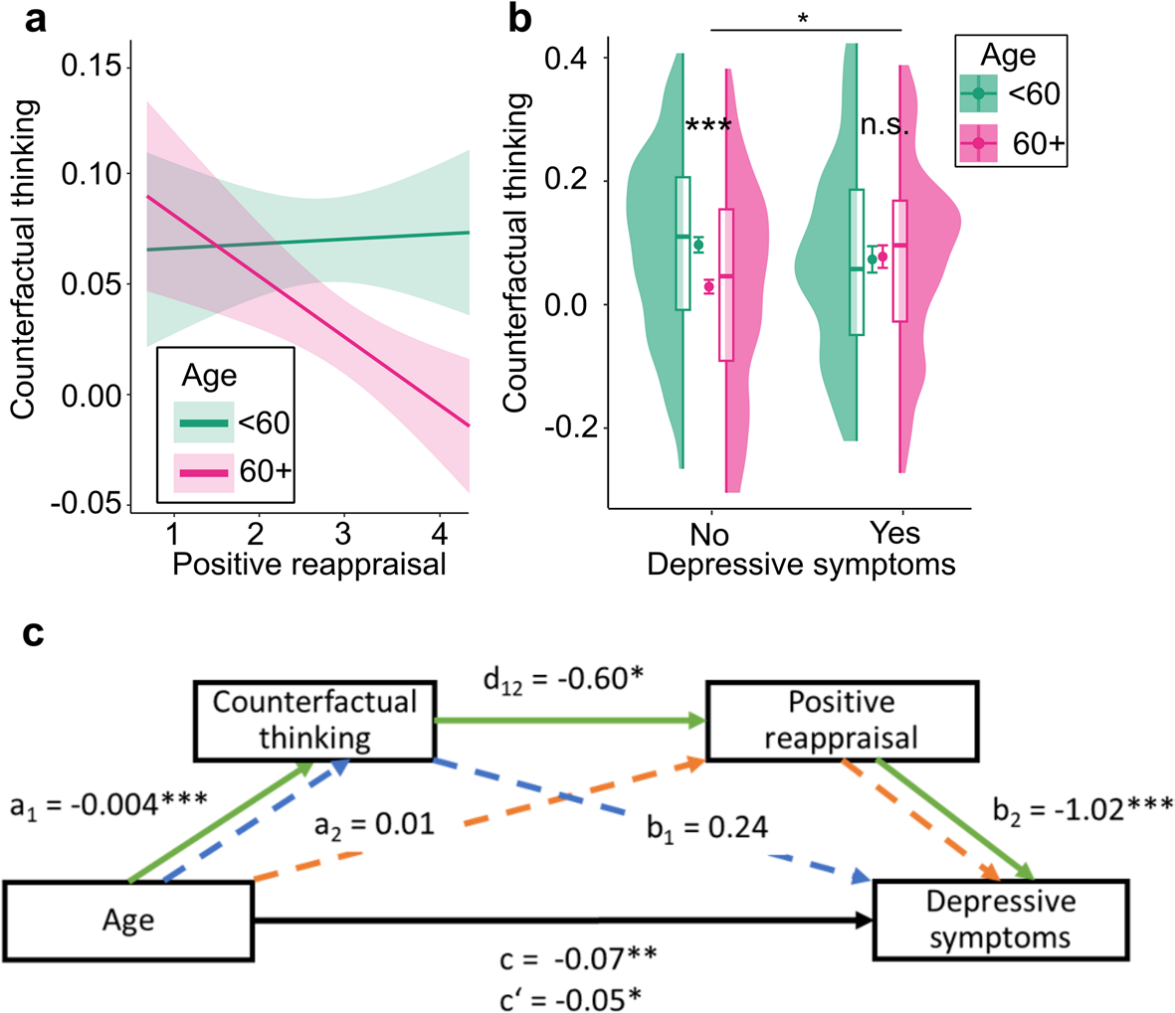
Age-specific relationships between CFT and emotional measures*.* (**a**) CFT correlates negatively with positive reappraisal only in older adults. (**b**) CFT is selectively lower in older adults without depressive symptoms. (**c**) Age-effects on depressive symptoms are partially mediated by serial effects of CFT and positive reappraisal (green pathway; dashed-line pathways are not significant). * *P* < 0.05, ** *P* < 0.005, *** *P* < 0.001

Across the whole sample, CFT was not directly related to brooding rumination (β = 0.04; *P* = 0.67), but there was a significantly negative correlation with positive reappraisal (β = −0.11; *P* = 0.013). As we expected an age-specific impact of CFT on emotion regulation strategies and indicators of emotional well-being, we repeated the analyses within age-subgroups (middle-aged: < 60, N = 195; older-aged: 60 +, N = 299). Results revealed that the negative relationship between CFT and positive reappraisal was solely driven by older adults (r_60+_  = −0.18, *P* = 0.002), but was not significant in middle-aged adults (r_<60_ = 0.03, *P* = 0.72). Group-interactions were significant (Fisher’s Z = 2.20, *P* = 0.014; Fig. 4a). No significant group-wise correlations emerged between CFT and brooding rumination (all *P* > 0.35).

Similarly, CFT was not directly related to life satisfaction or depressive symptoms across all participants (all *P* > 0.23). There was, however, a significant positive correlation between CFT and PHQ-scores in older adults (r_60+_  = 0.12, *P* = 0.04) but not middle-aged adults (r_<60_ = −0.06, *P* = 0.37), and this group-interaction was significant (Fisher’s Z = 1.97, *P* = 0.024). No substantial group-wise correlations emerged between CFT and life satisfaction (all *P* > 0.43).

To test the extension of our previous findings from old-young comparisons [8], we subdivided middle and older aged participants in those with (N_<60_ = 55, N_60+_  = 72) and without (N_<60_ = 140, N_60+_  = 227) significant depressive symptoms (cut-off point of 0–4 in PHQ-9 scores indicates no depressive symptoms, [30]) and tested group effects in predicting CFT using a univariate ANOVA. Results demonstrate a significant age by depression interaction (*F*(1,490) = 4.71; *P* = 0.03). That is, CFT was only decreased in non-depressed older adults while older adults with depressive symptoms showed significant CFT, very similar to middle-aged adults (Fig. 4b).

Finally, to integrate behavioral findings into a single model, a serial mediation analysis was applied to predict age-effects on depressive symptoms using CFT and positive reappraisal as serial mediators. Results revealed a significant serial effect of the pathway age—> CFT—> positive reappraisal—> depressive symptoms (*a*_*1*_*d*_*12*_*b*_*2*_ = −0.004; 95%-CI[−0.0101 −0.0003]; green pathways in Fig. 4c).

#### Counterfactual thinking and vmPFC resting-state functional connectivity

To characterize age-effects in vmPFC connectivity, we included age as parametric regressor in a seed-to-voxel functional connectivity analysis using a 10 mm sphere around vmPFC coordinates previously linked to regret-age interactions [8]. Across the whole sample, increasing age was associated with increasing vmPFC connectivity to the bilateral ventral striatum (putamen, caudatus) and the insula (frontal inferior operculum). vmPFC-connectivity was reduced with higher age with the bilateral medial and superior PFC, hippocampus, right amygdala, parietal-occipital regions as well as areas of the motor cortex and cerebellum (Supplementary Fig. 2, Supplementary Table 1).

**Fig.5:**
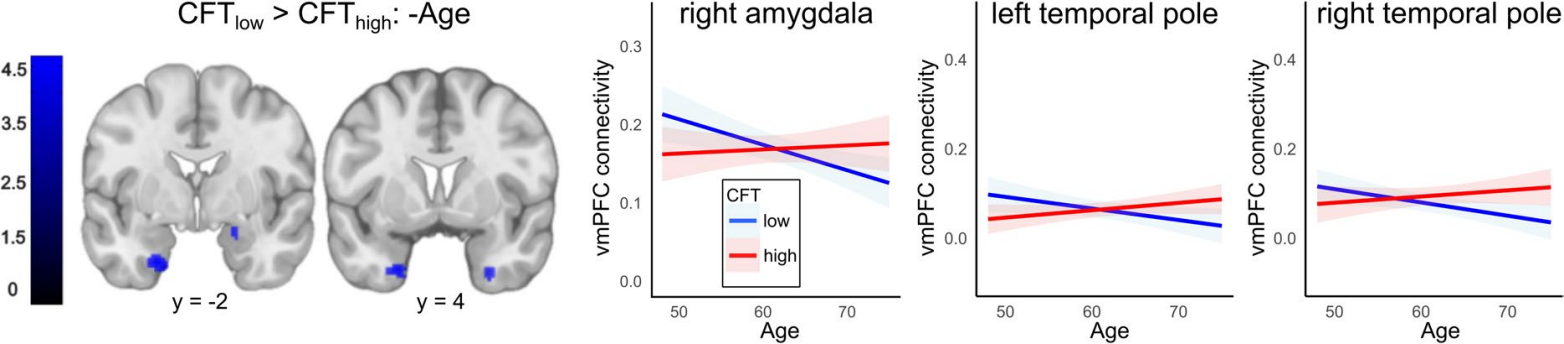
Differential age-trajectories for vmPFC connectivity in groups with high and low CFT. *P* < 0.05 FWE corrected

Next, we investigated whether age-slopes in vmPFC connectivity differ in groups with high versus low CFT. To this end, we used a median split on CFT to create a group factor (low vs. high CFT) and included age as parametric regressor. Results revealed a stronger age-related signal decrease in the low (versus high) CFT group in vmPFC connectivity with the bilateral temporal pole/entorhinal area and the right amygdala (all *P* < 0.05 FWE corrected, Fig. 5, Supplementary Table 2).

**Fig. 6:**
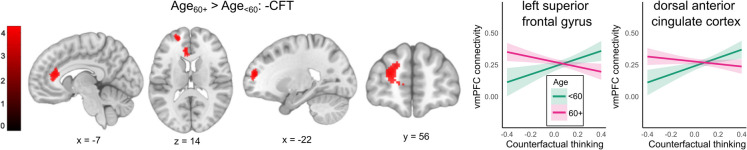
Age-specific relationship between vmPFC connectivity measures and CFT. *P* < 0.05 FWE corrected

Across the whole sample, CFT was not significantly correlated with vmPFC connectivity. However, comparing connectivity correlations with CFT between mid and older adults revealed significant effects in the superior prefrontal gyrus (sFG) and the dorsal anterior cingulate cortex (dACC) (all *P* < 0.05 FWE corrected, Fig. 6). While in middle age higher prefrontal connectivity was related to higher CFT, this pattern switched in older adults (all *P* < 0.05 FWE corrected; Fig. 6, Supplementary Table 2).

**Fig. 7:**
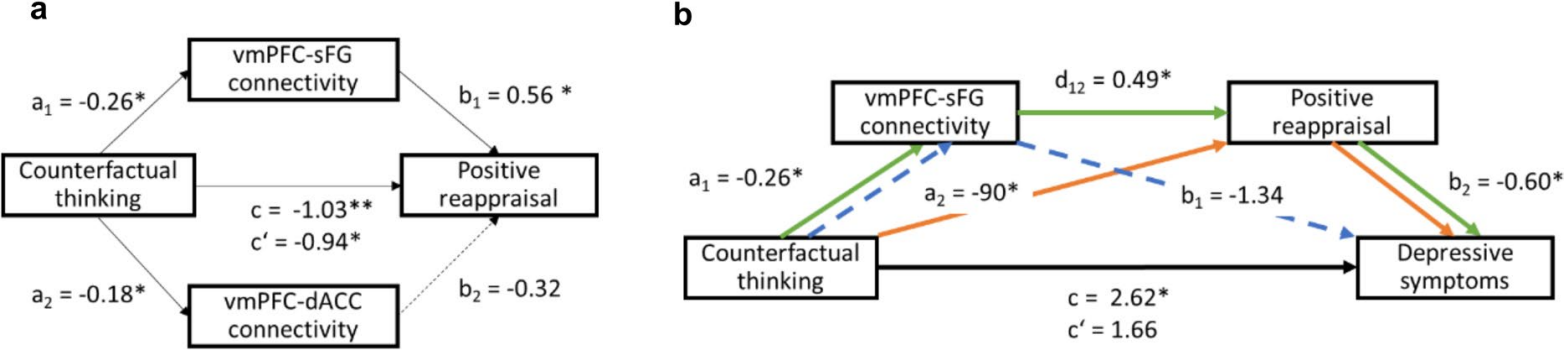
Mediation of positive effects of lower CFT on positive reappraisal (**a**) and emotional health (**b**) in older adults by vmPFC-sFG connectivity. * *P* < 0.05, ** *P* < 0.005

We then tested whether these networks are also involved in the critical relationship between CFT and positive reappraisal in older adults. Therefore, we conducted a parallel mediation analysis within older adults (60 +) with CFT as independent variable, positive reappraisal as dependent variable, and vmPFC-connectivity estimates from the sFG and the dACC as parallel mediators. Results revealed only vmPFC-sFG connectivity as a significant (partial) mediator (*a*_*1*_*b*_*1*_ = −0.14, 95%-CI[−0.36 −0.01], Fig. 7a).

Including vmPFC-sFG connectivity and positive reappraisal in a final serial mediation, indicated a fully mediated relationship between CFT and depressive symptoms in older adults by indirect effects from CFT—> sFG—> positive reappraisal—> depressive symptoms (*a*_*1*_*d*_*12*_*b*_*2*_ = 0.004, 95%-CI[0.0002 0.011]) in addition to indirect effects from CFT—> positive reappraisal—> depressive symptoms (*a*_*2*_*b*_*2*_ = 0.03, 95%-CI[0.004 0.06], Fig. 7b).

## Discussion

Our findings replicate previous epidemiological research suggesting a ‘paradox of aging’. In a population-based cohort of middle-aged to older adults, cross-sectional analyses show a linear positive relationship between age and life satisfaction, and a negative relationship between age and depressive symptoms. As a potential mechanism we found that experimentally assessed counterfactual thinking (CFT) was also lower at older ages compared to middle age, but only in individuals free of significant depressive symptoms.

In other words, while responsiveness to missed chances in middle age appears unrelated to affective state, it seems to become critical for well-being in older age. Results from mediation models suggest that reduced CFT supports emotional healthiness via facilitating a general positive focus as emotional regulation strategy. In older adults, the impact of reduced “what-if”-thinking on positively managing emotional conflicts in daily life was in turn specifically related to a stronger functional connectivity between the ventromedial and the dorsolateral prefrontal cortex. Thus, prefrontal connectivity findings point to (preserved) cognitive functioning as a potential resilience factor for adaptive emotion regulation in later life. The additional finding of reduced resting-state limbic connectivity in older adults with low CFT indicates an independent, positive side effect of age-related changes on emotional health.

Late-life depression (LLD) is more common than dementia and can have enormous socio-economic consequences, thus understanding the etiology is critical [16, 31]. A promising approach to investigate cognitive-emotional mechanisms and potential resilience factors is studying emotionally healthy aging. Epidemiological data consistently showed an increase in well-being during the second half of life [17, 18] and this held true even during the COVID-19 pandemic [32]. Here, we replicated this finding in a large population-based cohort both in terms of higher life-satisfaction and lower depressive symptoms in middle to old aged individuals, even when controlling for substantial covariates (gender, global cognition, partnership).

Our study is one of the first that combined the measurement of an experimentally controlled indicator of age-adapted decision making [8, 16] with the assessment of emotional well-being and a general positive regulation style across a continuous age-range. This enabled us to reveal a potential mechanism of mood stabilization in the daily life of older adults. Our findings of gradual differences in CFT from middle to older age indicate a change that may help older individuals to focus on positive aspects when dealing with daily challenges and thereby prevent negativity and depressive symptoms. Importantly, such an association was only obvious in later age (i.e., after 60). This supports the notion that at younger age, CFT may lead to active attempts to overcome regrets and thus optimization of future behavior [2]. However, as people age, less opportunities remain and less resources are available to correct past decisions (e.g., start a family, resolve conflict with someone who has already died) making disengagement from counterfactuals a more successful strategy [7, 33, 34].

Regret can be viewed as a subtype of upward counterfactual thinking with a dominant aspect of self-blame, whereas CFT per se can be neutral in terms of blame attributions [2] [35]. In accordance with this, CFT in our paradigm was not associated with depressive symptoms in middle age and not generally related to a ruminative response style. Thus, it cannot be considered as a “maladaptive” cognition per se but may become critical for emotional health when attributions of blame emerge and when simulated alternatives cannot (longer) be realized. Accordingly, compared to young adults, healthy elderly report lower levels of internal-control attributions [34], lower emotional intensity as well as lower intrusive thoughts associated with their potential real life-regrets [8].

It has been speculated that healthy older adults apply cognitive control strategies like external attribution to downregulate feelings of regret [34]. This assumption is supported by an increased vmPFC engagement in response to missed opportunities in task-based fMRI [8]. Moreover, studies involving cognitive manipulation support the relevance of cognitive resources and top-down regulation for age-related emotional adaptation [21, 29, 36, 37]. Yet, given the role of the vmPFC in implicit emotion regulation [24] its engagement may also reflect a shift towards automatic or implicit regulation [25, 38], triggered by life-long learning and limited resources [16]. Since the vmPFC is relatively preserved from age-related decline compared to more dorsolateral prefrontal areas [39] it has also been argued that vmPFC-related implicit and lower level regulation compensates for decline in higher order cognition [20]. Indeed, studies on early processes and habituation indicate that lower-level processes are involved in the emergence of emotional selectivity in older adults [40, 41]. Most likely, both automatic and flexible processes orchestrate age-related adaptation of emotional processing as a function of neurocognitive trade-offs, compensation, and lifelong learning.

The neuroimaging findings in the present study support such an interplay between higher- and lower-order processes in the favor of emotional resilience in aging. On the one hand, low levels of counterfactual thinking and positive reappraisal in aging was related to a higher connectivity of the vmPFC with the dlPFC. As part of the default mode network, the vmPFC is thought to contribute to monitoring and regulating internal mental states, processing self-related information, and supporting emotional and social cognition [42, 43]. The dlPFC plays a central role in the executive control network where it is crucial for executive functions including cognitive flexibility and conflict regulation [44]. The strength of functional connectivity between the executive control and default mode network decreases with age [45]. Furthermore, task-based fMRI studies implicate both the dlPFC and the vmPFC in emotion regulation (e.g. [24]). Thus, our findings indicate that preserved (intrinsic) functional connectivity within this network is crucial for an active disengagement from regret as individuals age. Notably, among middle-aged adults, stronger dlPFC connectivity was correlated with higher levels of CFT. The lack of association between CFT and depression in middle age suggests that individuals with strong cognitive resources may actively utilize CFT to optimize their behavior while letting go of potentially regretful thoughts as they age. In contrast, middle-aged adults with lower dlPFC resources may be more prone to engage in maladaptive CFT in later life.

On the other hand, we also observed a lower vmPFC connectivity with the amygdala and the temporal pole in older age, which was independently associated with reduced CFT. Lower vmPFC-amygdala resting state connectivity has been linked to better well-being and less negativity in older adults [46], while heightened resting state connectivity between the vmPFC and the amygdala has been related to self-focused rumination and greater emotional arousal in depression [47]. Resting-state connectivity of the temporal pole with the default mode network has been related to brooding, autobiographical memory, and aging [48, 49]. Future studies should investigate whether associations of these factors with CFT explain the temporal pole findings observed in the present study. Overall, our findings suggest that reduced vmPFC-limbic connectivity, as an epiphenomenon of healthy aging, may contribute to a reduced sensitivity to negative inputs and thoughts, such as regret.

While the large-scale sample investigated in this study is a significant strength, it also carries the potential for systematic biases, particularly concerning above-average cognitive and mental health status. To ensure accurate task performance, participants with significant global cognitive deficits (MMSE < 24) were excluded. Additionally, the prevalence of participants with strong depressive symptoms (PHQ-9 score ≥ 10) was lower than in German epidemiological surveys for this age group (e.g., 7.1% in [50], vs. 4.5% in the present study), which may limit the generalizability of our findings. Nevertheless, sufficient variability regarding subclinical depressive symptoms was present (with 25% having a PHQ-9 score ≥ 5), allowing us to explore key research questions related to potential risk factors for mood disturbance. Furthermore, given prior findings of increased CFT [8] and structural disruptions of fronto-subcortical networks [19] reported in late-life depression, it is conceivable that the differences observed here might be even more pronounced in a sample with a higher proportion of individuals with significant depressive symptoms.

The large-scale nature of this study also did not allow for task-based fMRI and thus for a direct comparison with target networks at rest within the same participant. Yet, it has been argued that functional networks that are continuously interacting with each other at rest to show a similar functional hierarchy that is seen during action and cognition [51]. We accounted for this by using a vmPFC seed that was empirically driven and reliant on past task-based data [8] and our findings strongly validate the crucial role of vmPFC networks in emotional resilience.

Additionally, due to time and logistical constraints inherent to large-scale studies like HCHS, only single-item assessments of emotion regulation and life satisfaction were feasible. While such measures have been successfully used in previous cohort studies [18], they inherently limit the granularity and validity of the emotional constructs assessed. Furthermore, the focus on the specific age range of HCHS limits the direct generalizability of the results to broader age groups and other definitions of midlife, which often span from approximately 40 to 60 years.

Finally, it should be noted that the cross-sectional design of our study limits the ability to make direct longitudinal inferences. Caution is therefore advised when interpreting the mediation models that include age [52]. Consequently, conclusions regarding age differences within individuals should be confirmed through longitudinal studies.

To our knowledge, the present study is the largest aging investigation into the neural correlates of counterfactual thinking conducted with an experimental task sensitive to regret responsiveness. Clinically, our findings regarding the increasing importance of CFT for emotional health from middle to late life speak to the importance of a time-critical intervention or prevention strategy. Equipping middle aged adults with appropriate techniques to deal with missed opportunities, such as cognitive restructuring and acceptance, might thereby be a promising approach to boost resilience against mood disorders in later life.

## Methods

### Sample

Data were obtained as part of the Hamburg City Health Study (HCHS), a prospective, population-based cohort study with a special focus on imaging and the general aim to identify individual predictors for and risk-factors of major chronic diseases (see [53] for rationale and design of the HCHS). Recruitment for the HCHS is performed via random sampling from the official inhabitant register to invite 45,000 inhabitants for study participation. Baseline assessments of data presented in this study took place between 2016 and 2020. The sample for the present study was obtained from the first 2,000 participants who underwent MRI scanning. In this cohort, a subsample of n = 612 completed the devil task and rsfMRI. After checking for data completeness and performing quality checks (see supplement for flow chart of exclusions), the final sample for this study comprised n = 494. The HCHS was approved by the local ethics committee (Hamburger Ärztekammer, PV5131, 27.10.2015) and written informed consent was obtained from all participants.

### Measures of emotional well-being

Participants reported on life satisfaction and depressive symptomatology. Specifically, life satisfaction was assessed with a single 11-point scale adapted from [18]: “*Please imagine a ladder with steps numbered from 0 at the bottom to 10 at the top. The top of the ladder represents the best possible life for you, and the bottom of the ladder represents the worst possible life for you. On which step of the ladder would you say you personally feel you stand at this time?*”. Using a single question to assess life satisfaction and global well-being is common and has been proven a reliable measure [54, 55]. Depressive symptoms were quantified by the sum-score of the nine-item Patient Health Questionnaire (PHQ-9, [30, 56]).

### Emotion regulation

Based on our hypotheses, we assessed two emotion-regulation styles/strategies in daily life: brooding rumination (adapted from Brans et al., [57]) and positive reappraisal (adapted from [58]). Specifically, participants were asked to indicate how they generally react when they feel sad or depressed (instruction adapted from the German version of the Response Style Questionnaire, RSQ, [59]) with a range from 1 (“very rarely”) to 4 (“almost always”) regarding the following two response styles: “I can’t stop thinking about my feelings” (brooding rumination) and “I try to find something positive in my situation” (positive reappraisal).

### Sequential risk-taking task (‘devil task’)

Participants performed 50 trials of a variant of a sequential risk-taking task (‘devil task’) [8, 28, 60]. On each trial participants were presented with a set of eight wooden boxes shown on a computer screen. By use of left and right arrow-keys on a standard keyboard, participants had to move a red frame to indicate until which box, starting from the left, the content of the boxes should be revealed. Seven of the boxes contained gold, whereas one box contained the devil. The aim was to maximize gains by opening boxes containing gold. One box of gold represented one point. If, however, the devil was revealed no points were collected for the present trial. Initial position of the red frame was set randomly at one of the eight boxes for each trial. If the frame had not been moved for 2 secs, the position was logged and boxes were opened until the indicated position or until the devil was exposed. In win trials, before the start of the next trial, the devil position was shown. Participants could thus see how many more boxes could have been safely opened (i.e., the missed chance on how many more gold could have been won). After a loss, participants could see how far their choice surpassed the devil position. Participants were asked to always move the frame, even if just back and forth, to ensure voluntary choice of the logged position. If the frame had not been moved for three trials in a row, a reminder to always move the frame appeared on the screen before the next trial. The task was programmed in Presentation® software and was presented on a computer at the study center of the HCHS. After receiving the automized task instructions participants performed a training phase of ten trials which was repeated up to five times until the frame had been moved in every trial.

To investigate the influence of prior task outcomes on subsequent risk-taking behavior we analyzed choices following win and loss trials separately. For each trial we calculated the difference between the devil position and the chosen position, representing outcome consequences (that is the magnitude of a missed chance for win trials and how much the chosen box surpassed the devil position for loss trials). We then performed regression analyses to predict the risk taken in trial t + 1 (the chosen number of boxes to open in the next trial) by the outcome consequences (the devil-minus-choice difference for win trials and vice versa for lost trials to equalize direction) of the present trial t (Eq. 1, Eq. 2).

Ct_win trials:

1 $$Risk taking\left(t+1\right)={\beta }_{0}+{\beta }_{1}\times \left(devil position \left(t\right)-choice position\left(t\right)\right), t=win trial$$

Ct_lost trials:

2 $$Risk taking\left(t+1\right)={\beta }_{0}+{\beta }_{1}\times \left(choice position\left(t\right)-devil position\left(t\right)\right), t=lost trial$$

### MRI acquisition

Images were acquired on a 3 T whole MRI scanner (Siemens Skyra, Siemens Erlangen, Germany). Detailed procedural information can be found in the previously published study protocol [53]. Anatomical T1-weighted images were acquired using a rapid acquisition gradient-echo sequence (MPRAGE, sequence parameter: TR = 2500 ms, TE = 2.12 ms, 256 axial slices, slice thickness = 0.94 mm, and in-plane resolution = 0.83 × 0.83 mm2). Resting-state fMRI scans were acquired using an echo-planar-imaging sequence (sequence parameter: TR = 3000 ms, TE = 32 ms, FOV = 192 × 192 mm2, 46 slices, flip angle = 90°, matrix = 64 × 64, slice thickness = 3 mm, slice gap = 0 mm, effective voxel resolution = 3.0 × 3.0 × 3.0 mm3).

### Functional MRI data analysis

Functional connectivity analyses were carried out using CONN-toolbox version 21a (RRID: SCR_009550 www.nitric.org/projects/conn, [61]). Following the default data-preprocessing pipeline of the toolbox, functional realignment, slice-time correction, structural segmentation and normalization to the Montreal Neurological Institute (MNI) template, functional segmentation and normalization and smoothing (8 mm full-width at half maximum Gaussian kernel) were applied. Denoising of the blood oxygenation level-dependent (BOLD) signal to remove physiological and other sources of noise was performed using the CompCor method [62] as implemented in the toolbox. In detail, a white matter component, a cerebrospinal fluid component and a realignment confound (as obtained from the estimated motion parameters consisting of six rigid-body dimensions plus their first-order derivatives) were regressed from the BOLD signal [63]. Outliers were identified based on the intermediate motion threshold defined in the toolbox corresponding to the global mean signal intensity exceeding 5 SD or slice to slice movement exceeding 0.9 mm. As part of denoising, scrubbing of outliers was applied using the Artifact detection toolbox within CONN (ART, http://www.nitrc.org/projects/artifact_detect/) by regressing out noise components for identified outlier scans. Band-pass filtering (0.008–0.09 Hz) was then applied to the residual BOLD time series.

We then computed seed-to-voxel correlation maps on the first level (within-subjects). In detail, Pearson correlations between the mean residual BOLD time series of the seed region and the time series of all other voxels in the brain were computed and transformed using Fisher’s r-to-z transformation. Ventromedial PFC seed was defined as 10 mm sphere centered on MNI x, y,z −4, 44, −10 [8]. First-level connectivity maps were then entered into a whole-brain analysis at the group level. To model behavior interactions on functional connectivity, we specified a regression model with age as covariate as well as two ANCOVA models: one using age as group factor and CFT as covariate and one with CFT as group factor and age as covariate. We report results corrected for family wise errors (FWE < 0.05) due to multiple comparisons. Correction was conducted at the cluster level after passing a cluster forming threshold of P < 0.001 uncorrected and at the peak voxel within bilateral anatomical masks (AAL3 atlas; [64]) of the amygdala and the nucleus accumbens (NAc) given our main interest in the role of prefrontal-limbic interactions.

### Behavioral data analysis/Statistical analysis

Simple and multiple regression models were used to analyze age effects in our behavioral variables. Mediation analyses on behavioral and neural measures were performed using Hayes' PROCESS macro v4.2 for R [65], which uses ordinary least squares regressions, yielding unstandardized and standardized path coefficients for total, direct, and indirect effects. To test our mediation hypotheses, simple, parallel, and serial mediation models were conducted using Model 4 and 6. Bootstrapping with 5000 samples together with heteroscedasticity consistent standard errors were employed to compute 95% confidence intervals (CIs) and inferential statistics. All analyses were conducted using R and all statistical tests were two-sided with P < 0.05 used to indicate statistical significance.

## Supplementary Information

Below is the link to the electronic supplementary material.

Supplementary file1 (DOCX 884 KB)

## Data Availability

Behavioral and imaging data that support the findings of this study have been deposited online under the following link: 10.6084/m9.figshare.28638908.

## References

[1] Broomhall AG, Phillips WJ, Hine DW, Loi NM. Upward counterfactual thinking and depression: a meta-analysis. Clin Psychol Rev. 2017;55:56–73. 10.1016/j.cpr.2017.04.010. 28501706 10.1016/j.cpr.2017.04.010

[2] Byrne RMJ. Counterfactual thought. Annu Rev Psychol. 2016;67:135–57. 10.1146/annurev-psych-122414-033249. 26393873 10.1146/annurev-psych-122414-033249

[3] Kahneman D, Slovic SP, Slovic P, Tversky A. Judgment Under Uncertainty: Heuristics and Biases. Cambridge University Press; 1982. 10.1126/science.185.4157.1124 17835457

[4] Wrosch C, Bauer I, Scheier MF. Regret and quality of life across the adult life span: the influence of disengagement and available future goals. Psychol Aging. 2005;20(4):657–70. 10.1037/0882-7974.20.4.657. 16420140 10.1037/0882-7974.20.4.657

[5] Ingersoll-Dayton B, Torges C, Krause N. Unforgiveness, rumination, and depressive symptoms among older adults. Aging Ment Health. 2010;14(4):439–49. 10.1080/13607860903483136. 20455120 10.1080/13607860903483136 PMC2868276

[6] Baltes P, Baltes M. Successful aging: Perspectives from the behavioral sciences. Cambridge University Press; 1990.

[7] Heckhausen J, Wrosch C, Schulz R. A motivational theory of life-span development. Psychol Rev. 2010;117(1):32–60. 10.1037/a0017668. 20063963 10.1037/a0017668 PMC2820305

[8] Brassen S, Gamer M, Peters J, Gluth S, Büchel C. Don’t look back in anger! Responsiveness to missed chances in successful and nonsuccessful aging. Science. 2012;336(6081):612–4. 10.1126/science.1217516. 22517323 10.1126/science.1217516

[9] Sütterlin S, Paap MCS, Babic S, Kübler A, Vögele C. Rumination and age: some things get better. J Aging Res. 2012;2012:267327. 10.1155/2012/267327. 22500227 10.1155/2012/267327 PMC3303571

[10] Epstude K, Roese NJ. The functional theory of counterfactual thinking. Pers Soc Psychol Rev. 2008;12(2):168. 10.1177/1088868308316091. 18453477 10.1177/1088868308316091 PMC2408534

[11] Nowlan JS, Wuthrich VM, Rapee RM. Positive reappraisal in older adults: a systematic literature review. Aging Ment Health. 2015;19(6):475–84. 10.1080/13607863.2014.954528. 25204368 10.1080/13607863.2014.954528

[12] Ferguson HJ, Brunsdon VEA, Bradford EEF. The developmental trajectories of executive function from adolescence to old age. Sci Rep. 2021. 10.1038/s41598-020-80866-1. 33446798 10.1038/s41598-020-80866-1 PMC7809200

[13] Gunning-Dixon FM, Brickman AM, Cheng JC. Aging of cerebral white matter: a review of MRI findings. Int J Geriatr Psychiatry. 2009;24(2):109–17. 10.1002/gps.2087. 18637641 10.1002/gps.2087 PMC2631089

[14] Park DC, Festini SB (2016) The Middle-Aged Brain: A Cognitive Neuroscience Perspective. In R. Cabeza, L. Nyberg, & D. C. Park (Eds.), *Cognitive Neuroscience of Aging: Linking Cognitive and Cerebral Aging* (p. 0). Oxford University Press. 10.1093/acprof:oso/9780199372935.003.0015

[15] Richmond-Rakerd LS, Caspi A, Ambler A, d’Arbeloff T, de Bruine M, Elliott M, et al. Childhood self-control forecasts the pace of midlife aging and preparedness for old age. Proc Natl Acad Sci U S A. 2021;118(3):e2010211118. 10.1073/pnas.2010211118. 33397808 10.1073/pnas.2010211118 PMC7826388

[16] Thams F, Brassen S. The need to change: is there a critical role of midlife adaptation in mental health later in life? Elife. 2023;12:e82390. 10.7554/eLife.82390. 37141113 10.7554/eLife.82390 PMC10159621

[17] Blanchflower DG. Is happiness U-shaped everywhere? Age and subjective well-being in 145 countries. J Popul Econ. 2021;34(2):575–624. 10.1007/s00148-020-00797-z. 32929308 10.1007/s00148-020-00797-z PMC7480662

[18] Stone AA, Schwartz JE, Broderick JE, Deaton A. A snapshot of the age distribution of psychological well-being in the United States. Proc Natl Acad Sci U S A. 2010;107(22):9985–90. 10.1073/pnas.1003744107. 20479218 10.1073/pnas.1003744107 PMC2890490

[19] Kim Y-K, Han K-M. Neural substrates for late-life depression: a selective review of structural neuroimaging studies. Prog Neuropsychopharmacol Biol Psychiatry. 2021;104:110010. 10.1016/j.pnpbp.2020.110010. 32544600 10.1016/j.pnpbp.2020.110010

[20] Mather M. The affective neuroscience of aging. Annu Rev Psychol. 2016;67:213–38. 10.1146/annurev-psych-122414-033540. 26436717 10.1146/annurev-psych-122414-033540 PMC5780182

[21] Brassen S, Gamer M, Büchel C. Anterior cingulate activation is related to a positivity bias and emotional stability in successful aging. Biol Psychiatry. 2011;70(2):131–7. 21183158 10.1016/j.biopsych.2010.10.013

[22] Corbett B, Rajah MN, Duarte A. Preparing for the worst: evidence that older adults proactively downregulate negative affect. Cereb Cortex. 2020;30(3):1291–306. 10.1093/cercor/bhz166. 31424075 10.1093/cercor/bhz166 PMC8205626

[23] Leclerc CM, Kensinger EA. Neural processing of emotional pictures and words: a comparison of young and older adults. Dev Neuropsychol. 2011;36(4):519–38. 10.1080/87565641.2010.549864. 21516546 10.1080/87565641.2010.549864

[24] Etkin A, Büchel C, Gross JJ. The neural bases of emotion regulation. Nat Rev Neurosci. 2015;16(11):693–700. 10.1038/nrn4044. 26481098 10.1038/nrn4044

[25] Suri G, Gross JJ. Emotion regulation and successful aging. Trends Cogn Sci. 2012;16(8):409–10. 10.1016/j.tics.2012.06.007. 22739000 10.1016/j.tics.2012.06.007

[26] Cacioppo JT, Berntson GG, Bechara A, Tranel D, Hawkley LC (2011) Could an aging brain contribute to subjective well-being? The value added by a social neuroscience perspective. In A. Todorov, S. Fiske, & D. Prentice (Eds.), *Social Neuroscience: Toward Understanding the Underpinnings of the Social Mind* (p. 0). Oxford University Press. 10.1093/acprof:oso/9780195316872.003.0017

[27] Mather M. The emotion paradox in the aging body and brain. Ann N Y Acad Sci. 2024. 10.1111/nyas.15138. 38676452 10.1111/nyas.15138

[28] Büchel C, Brassen S, Yacubian J, Kalisch R, Sommer T. Ventral striatal signal changes represent missed opportunities and predict future choice. Neuroimage. 2011;57(3):1124–30. 10.1016/j.neuroimage.2011.05.031. 21616154 10.1016/j.neuroimage.2011.05.031

[29] Mather M. The emotion paradox in the aging brain. Ann N Y Acad Sci. 2012;1251:33–49. 10.1111/j.1749-6632.2012.06471.x. 22409159 10.1111/j.1749-6632.2012.06471.x PMC3395773

[30] Kroenke K, Spitzer RL, Williams JB. The PHQ-9: validity of a brief depression severity measure. J Gen Intern Med. 2001;16(9):606–13. 10.1046/j.1525-1497.2001.016009606.x. 11556941 10.1046/j.1525-1497.2001.016009606.x PMC1495268

[31] Naismith SL, Norrie LM, Mowszowski L, Hickie IB. The neurobiology of depression in later-life: clinical, neuropsychological, neuroimaging and pathophysiological features. Prog Neurobiol. 2012;98(1):99–143. 10.1016/j.pneurobio.2012.05.009. 22609700 10.1016/j.pneurobio.2012.05.009

[32] Carstensen LL, Shavit YZ, Barnes JT. Age advantages in emotional experience persist even under threat from the COVID-19 pandemic. Psychol Sci. 2020;31(11):1374–85. 10.1177/0956797620967261. 33104409 10.1177/0956797620967261 PMC13171095

[33] Lecci L, Okun MA, Karoly P. Life regrets and current goals as predictors of psychological adjustment. J Pers Soc Psychol. 1994;66(4):731–41. 10.1037/0022-3514.66.4.731.

[34] Wrosch C, Heckhausen J. Perceived control of life regrets: good for young and bad for old adults. Psychol Aging. 2002;17(2):340–50. 12061416

[35] Mellers B, Schwartz A, Ritov I. Emotion-based choice. J Exp Psychol Gen. 1999;128(3):332–45. 10.1037/0096-3445.128.3.332.

[36] Kryla-Lighthall N, Mather M (2009) The role of cognitive control in older adults’ emotional well-being. In *Handbook of theories of aging, 2nd ed* (pp. 323–344). Springer Publishing Company

[37] Sasse LK, Gamer M, Büchel C, Brassen S. Selective control of attention supports the positivity effect in aging. PLoS ONE. 2014;9(8):e104180. 10.1371/journal.pone.0104180. 25093459 10.1371/journal.pone.0104180 PMC4122404

[38] Isaacowitz DM. What do we know about aging and emotion regulation? Perspect Psychol Sci. 2022;17(6):1541–55. 10.1177/17456916211059819. 35605229 10.1177/17456916211059819 PMC9633333

[39] Fjell AM, Westlye LT, Amlien I, Espeseth T, Reinvang I, Raz N, et al. High consistency of regional cortical thinning in aging across multiple samples. Cereb Cortex. 2009;19(9):2001–12. 10.1093/cercor/bhn232. 19150922 10.1093/cercor/bhn232 PMC2733683

[40] Gronchi G, Righi S, Pierguidi L, Giovannelli F, Murasecco I, Viggiano MP. Automatic and controlled attentional orienting in the elderly: a dual-process view of the positivity effect. Acta Psychol. 2018;185:229–34. 10.1016/j.actpsy.2018.02.008. 10.1016/j.actpsy.2018.02.008 29550693

[41] Petro NM, Basyouni R, Neta M. Positivity effect in aging: evidence for the primacy of positive responses to emotional ambiguity. Neurobiol Aging. 2021;106:232–40. 10.1016/j.neurobiolaging.2021.06.015. 34311432 10.1016/j.neurobiolaging.2021.06.015 PMC9255668

[42] Andrews-Hanna JR, Reidler JS, Huang C, Buckner RL. Evidence for the default network’s role in spontaneous cognition. J Neurophysiol. 2010;104(1):322–35. 10.1152/jn.00830.2009. 20463201 10.1152/jn.00830.2009 PMC2904225

[43] Roy M, Shohamy D, Wager TD. Ventromedial prefrontal-subcortical systems and the generation of affective meaning. Trends Cogn Sci. 2012;16(3):3. 10.1016/j.tics.2012.01.005. 22310704 10.1016/j.tics.2012.01.005 PMC3318966

[44] Seeley WW, Menon V, Schatzberg AF, Keller J, Glover GH, Kenna H, et al. Dissociable intrinsic connectivity networks for salience processing and executive control. J Neurosci. 2007;27(9):2349–56. 10.1523/JNEUROSCI.5587-06.2007. 17329432 10.1523/JNEUROSCI.5587-06.2007 PMC2680293

[45] Chrysikou EG, Caulfield MD, Kan IP. Large-scale network connectivity as a predictor of age: evidence across the adult lifespan from the Cam-CAN data set. Psychol Aging. 2022;37(5):557–74. 10.1037/pag0000683. 35604697 10.1037/pag0000683

[46] Pruitt PJ, Yu K, Lahna D, Schwartz D, Peltier SJ, Silbert LC, et al. Emotional characteristics associate with fronto-amygdala connectivity in older-old adults: results from I-CONECT. Alzheimers Dement. 2023;19(S5):e067142. 10.1002/alz.067142.

[47] Burghy CA, Stodola DE, Ruttle PL, Molloy EK, Armstrong JM, Oler JA, et al. Developmental pathways to amygdala-prefrontal function and internalizing symptoms in adolescence. Nat Neurosci. 2012;15(12):1736–41. 10.1038/nn.3257. 23143517 10.1038/nn.3257 PMC3509229

[48] Kim J, Andrews-Hanna JR, Eisenbarth H, Lux BK, Kim HJ, Lee E, et al. A dorsomedial prefrontal cortex-based dynamic functional connectivity model of rumination. Nat Commun. 2023;14(1):3540. 10.1038/s41467-023-39142-9. 37321986 10.1038/s41467-023-39142-9 PMC10272121

[49] Setton R, Mwilambwe-Tshilobo L, Sheldon S, Turner GR, Spreng RN. Hippocampus and temporal pole functional connectivity is associated with age and individual differences in autobiographical memory. Proc Natl Acad Sci U S A. 2022;119(41):e2203039119. 10.1073/pnas.2203039119. 36191210 10.1073/pnas.2203039119 PMC9564102

[50] Busch MA, Maske UE, Ryl L, Schlack R, Hapke U. Prevalence of depressive symptoms and diagnosed depression among adults in Germany: Results of the German Health Interview and Examination Survey for Adults (DEGS1). Bundesgesundheitsblatt Gesundheitsforschung Gesundheitsschutz. 2013;56(5–6):733–9. 10.1007/s00103-013-1688-3. 23703492 10.1007/s00103-013-1688-3

[51] Tavor I, Jones OP, Mars RB, Smith SM, Behrens TE, Jbabdi S. Task-free MRI predicts individual differences in brain activity during task performance. Science. 2016;352(6282):216–20. 10.1126/science.aad8127. 27124457 10.1126/science.aad8127 PMC6309730

[52] Lindenberger U, von Oertzen T, Ghisletta P, Hertzog C. Cross-sectional age variance extraction: what’s change got to do with it? Psychol Aging. 2011;26(1):34–47. 10.1037/a0020525. 21417539 10.1037/a0020525

[53] Jagodzinski A, Johansen C, Koch-Gromus U, Aarabi G, Adam G, Anders S, et al. Rationale and design of the Hamburg City Health Study. Eur J Epidemiol. 2020;35(2):169–81. 10.1007/s10654-019-00577-4. 31705407 10.1007/s10654-019-00577-4 PMC7125064

[54] Diener E, Oishi S, Tay L. Advances in subjective well-being research. Nat Hum Behav. 2018;2(4):253–60. 10.1038/s41562-018-0307-6. 30936533 10.1038/s41562-018-0307-6

[55] Schimmack U, Oishi S. The influence of chronically and temporarily accessible information on life satisfaction judgments. J Pers Soc Psychol. 2005;89(3):395–406. 10.1037/0022-3514.89.3.395. 16248721 10.1037/0022-3514.89.3.395

[56] Löwe B, Spitzer RL, Gräfe K, Kroenke K, Quenter A, Zipfel S, et al. Comparative validity of three screening questionnaires for DSM-IV depressive disorders and physicians’ diagnoses. J Affect Disord. 2004;78(2):131–40. 10.1016/S0165-0327(02)00237-9. 14706723 10.1016/s0165-0327(02)00237-9

[57] Brans K, Koval P, Verduyn P, Lim YL, Kuppens P. The regulation of negative and positive affect in daily life. Emotion (Washington, DC). 2013;13(5):926–39. 10.1037/a0032400. 23731436 10.1037/a0032400

[58] Carver CS. You want to measure coping but your protocol’s too long: consider the brief COPE. Int J Behav Med. 1997;4(1):92–100. 10.1207/s15327558ijbm0401_6. 16250744 10.1207/s15327558ijbm0401_6

[59] Kühner C, Huffziger S, Nolen-Hoeksema S (2007) *Response styles questionnaire: RSQ-D ; deutsche Version*. Hogrefe

[60] Slovic P. Risk-taking in children: age and sex differences. Child Dev. 1966;37(1):169. 10.2307/1126437.

[61] Whitfield-Gabrieli S, Nieto-Castanon A. Conn: a functional connectivity toolbox for correlated and anticorrelated brain networks. Brain Connect. 2012;2(3):125–41. 22642651 10.1089/brain.2012.0073

[62] Behzadi Y, Restom K, Liau J, Liu TT. A component based noise correction method (CompCor) for BOLD and perfusion based fMRI. Neuroimage. 2007;37(1):90–101. 17560126 10.1016/j.neuroimage.2007.04.042 PMC2214855

[63] Power JD, Mitra A, Laumann TO, Snyder AZ, Schlaggar BL, Petersen SE. Methods to detect, characterize, and remove motion artifact in resting state fMRI. Neuroimage. 2014;84:320–41. 10.1016/j.neuroimage.2013.08.048. 23994314 10.1016/j.neuroimage.2013.08.048 PMC3849338

[64] Rolls ET, Huang C-C, Lin C-P, Feng J, Joliot M. Automated anatomical labelling atlas 3. Neuroimage. 2020;206:116189. 10.1016/j.neuroimage.2019.116189. 31521825 10.1016/j.neuroimage.2019.116189

[65] Hayes AF, Rockwood NJ. Conditional process analysis: concepts, computation, and advances in the modeling of the contingencies of mechanisms. Am Behav Sci. 2020;64(1):19–54. 10.1177/0002764219859633.

