# Supplementary material for: Age differences in what-if thinking from midlife onwards: Prefrontal contribution and implications for emotional health in late life
Source: GeroScience. 2025 Oct 7;48(3):4431–45. doi: 10.1007/s11357-025-01928-8 (PMC13356248; doi:10.1007/s11357-025-01928-8)
Supplement: Supplementary file 1 — Supplementary file1 (DOCX 884 KB) [file 11357_2025_1928_MOESM1_ESM.docx]

## Supplementary results


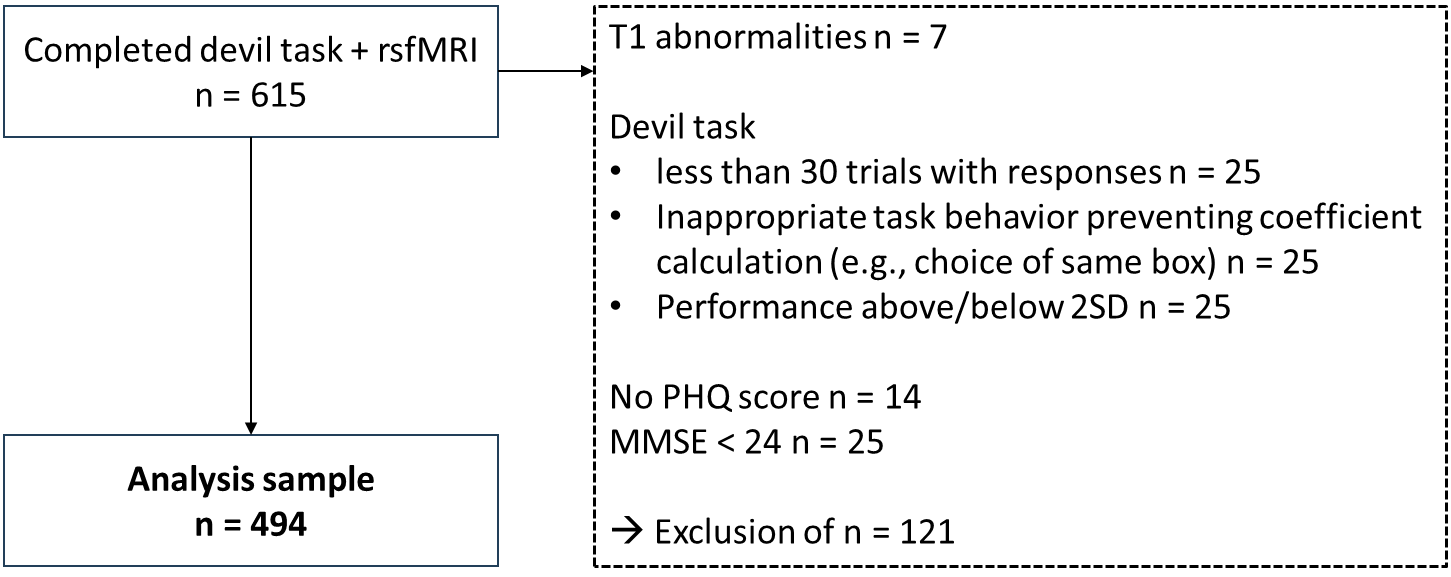


**Supplementary Fig. 1 |** Study flow chart.


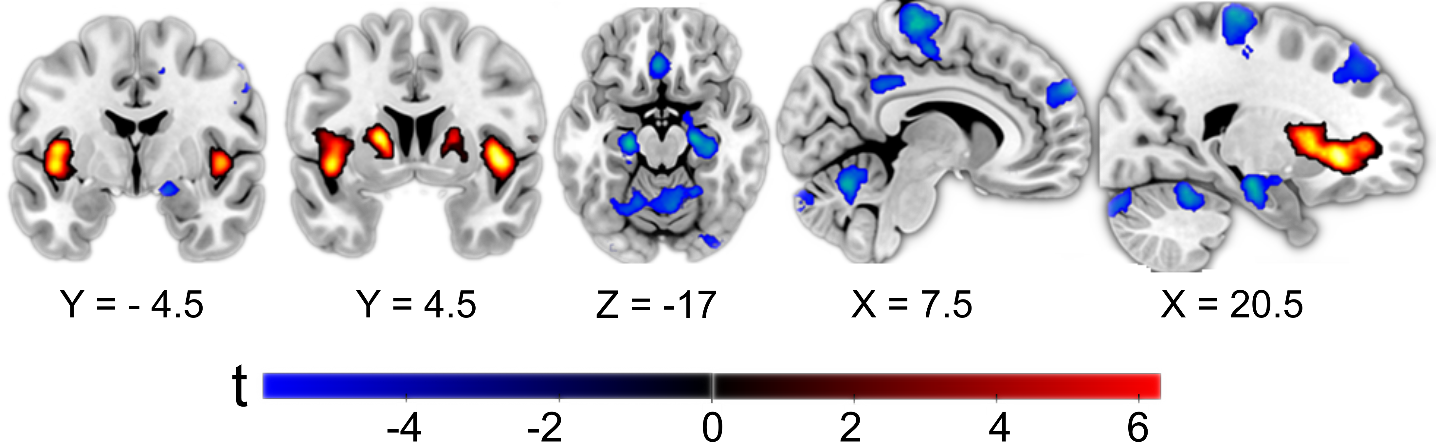


**Supplementary Fig. 2 |** Age-effects in resting state vmPFC connectivity. Red indicates an age-related increase, blue indicates an age-related decrease.

|  |  | MNI (peak) | | |  |  |
| --- | --- | --- | --- | --- | --- | --- |
| Brain region | Side | x | y | z | Cluster size | Z |
| **Main effect age** |  |  |  |  |  |  |
| *-age* |  |  |  |  |  |  |
| Supplementary motor area | R | 4 | -20 | 68 | 4379 | 5.78 |
| Hippocampus | L | ‐22 | ‐22 | ‐16 | 387 | 5.55 |
|  | R | 22 | 18 | -18 | 524 | 5.25 |
| Subgenual ACC | L | -2 | 32 | -10 | 1688 | 5.47 |
| Cerebellum | R | 10 | -62 | -22 | 1462 | 5.20 |
|  | L | -26 | -90 | -28 | 405 | 4.79 |
| Angular gyrus | L | -54 | -66 | 40 | 277 | 5.13 |
|  | R | 60 | -58 | 30 | 333 | 4.77 |
| Posterior cingulate gyrus | R | 12 | -40 | 34 | 710 | 4.84 |
| Postcentral gyrus | L | -48 | -14 | 38 | 221 | 4.28 |
| Inferior occipital gyrus | L | -30 | -84 | 2 | 579 | 4.26 |
| Superior frontal gyrus | R | 22 | 36 | 46 | 302 | 4.09 |
|  | L | -22 | 40 | 44 | 148 | 4.04 |
| Amygdala | R | 18 | -2 | -14 | 7 | 3.78* |
|  |  |  |  |  |  |  |
| *age* |  |  |  |  |  |  |
| Caudatus | L | -20 | 22 | 0 | 2819 | 6.18 |
| Putamen | L | -18 | 4 | 10 | same cluster | 5.64 |
| Insula | L | -44 | 2 | -4 | same cluster | 5.58 |
| Caudatus | R | 22 | 26 | 2 | 2131 | 5.98 |
| Insula | R | 44 | 4 | -2 | same cluster | 5.68 |
| Putamen | R | 22 | 12 | 6 | same cluster | 5.45 |
| Inferior frontal gyrus | R | 52 | 42 | 8 | 206 | 4.32 |
|  |  |  |  |  |  |  |
|  | | | | | | |
| **Supplementary Table 1 \| Peak coordinates and statistics for rsfMRI activations**  Montreal Neurological Institute (MNI) coordinates and the respective z-values are reported for peak voxels and local maxima within each cluster. All p < .05 FWE corrected. Significant results based on whole-brain correction or small volume corrections (*) within our regions of interest (bilateral Amygdala, bilateral NAc). L: left, R: right, n.s.: not significant | | | | | | |

|  |  | MNI (peak) | | |  |  |
| --- | --- | --- | --- | --- | --- | --- |
| Brain region | Side | x | y | z | Cluster size | Z |
| **Age effects in CT_low_ vs. CT_high_** |  |  |  |  |  |  |
| *CT_low_ > CT_high_: -age* |  |  |  |  |  |  |
| Temporal Pole | R | 28 | 10 | -44 | 148 | 4.61 |
|  | L | ‐24 | ‐2 | ‐36 | 147 | 4.29 |
| Amygdala | R | 20 | -2 | -18 | 27 | 3.35* |
|  |  |  |  |  |  |  |
| *CT_low_ > CT_high_: age* |  |  |  |  |  |  |
| n.s. |  |  |  |  |  |  |
|  |  |  |  |  |  |  |
| **CFT correlates in Age_60+_ vs. Age_<60_** |  |  |  |  |  |  |
| *Age_60+_ > Age_<60_: -CFT* |  |  |  |  |  |  |
| Superior frontal gyrus (dlPFC) | L | ‐20 | 52 | 16 | 195 | 4.19 |
| dACC | L | -8 | 32 | 16 | 157 | 4.01 |
|  |  |  |  |  |  |  |
| *Age_60+_ > Age_<60_: CFT* |  |  |  |  |  |  |
| n.s |  |  |  |  |  |  |
|  |  |  |  |  |  |  |
|  | | | | | | |
| **Supplementary Table 2 \| Peak coordinates and statistics for fMRI activations**  Montreal Neurological Institute (MNI) coordinates and the respective z-values are reported for peak voxels and local maxima within each cluster. All p < .05 FWE corrected. Significant results based on whole-brain correction or small volume corrections (*) within our regions of interest (bilateral Amygdala, bilateral NAc). L: left, R: right, n.s.: not significant | | | | | | |
